# On “the remedies and medications which were given to ailing negroes:” health and death spending on slaves by the Office of the Jesuit Province of Paraguay, 1711-1745

**DOI:** 10.1590/S0104-59702023000100036

**Published:** 2023-08-11

**Authors:** Eliane Cristina Deckmann Fleck, Antonio Dari Ramos

**Affiliations:** i Professora visitante, Programa de Pós-graduação em História/Universidade Federal de Pelotas. Pelotas – RS – Brasil ecdfleck@gmail.com; i Professor associado IV, Faculdade Intercultural Indígena e Cátedra Unesco Gênero, Diversidade Cultural e Fronteiras/Universidade Federal da Grande Dourados. Dourados – MS – Brasil antonioramos@ufgd.edu.br

**Keywords:** Company of Jesus, Office of the Jesuit Province of Paraguay, Slaves, Disease, Death, Companhia de Jesus, Ofício da Província Jesuítica do Paraguay, Escravos, Doenças, Morte

## Abstract

This text analyzes the way sick slaves were treated at the Office (ofício) of the Jesuit Province of Paraguay and Santa Catalina Farm (estancia) between 1711 and 1745. The sources consulted – Libro de cuentas del Ofício, Memoriales, and Cartas ânuas – reveal that the sickness of the enslaved people generated expenses, not only for medications, clothing, and food, but also for shrouds for their burial. As for the slaves from the Santa Catalina Farm, the sources indicate that depending on the infirmity, they were sometimes sent to Córdoba, where they were treated by laypersons trained in the healing arts, which incurred different expenses, also recorded in the ledgers.

## Abstract (pt)

Neste texto, analisamos o tratamento dispensado aos escravos enfermos do Ofício da Província Jesuítica do Paraguay e da Estância de Santa Catalina entre 1711 e 1745. As fontes analisadas – *Libro de cuentas del Ofício, Memoriales* e *Cartas ânuas* – revelam que o adoecimento de escravos do Ofício gerava despesas tanto com a aquisição de medicamentos, roupas e alimentos quanto com mortalhas para seu sepultamento. No caso dos escravos da Estância de Santa Catalina, observou-se que, dependendo da enfermidade, eles eram encaminhados à cidade de Córdoba, onde eram tratados por leigos treinados nas artes de curar, o que implicava despesas de outra natureza também registradas nos relatórios contábeis.

Na época colonial, a cidade de Córdoba, na atual Argentina, foi um importante centro de compra e venda de mão de obra escrava, um ponto nodal das redes de distribuição escravista para o Chile e para as minas de Potosi, interligando-se, via Buenos Aires, aos mercadores de escravos do Rio de Janeiro. No final do século XVIII, a quantidade de escravos chegava a 29,5% da população da cidade (Celton et al., 2020, p.115), e boa parte deles pertencia à Companhia de Jesus, trabalhando nas suas propriedades rurais e urbanas.

Os escravos utilizados nas propriedades jesuítas de Córdoba, como indicam os *Livros de contas* e as *Cartas ânuas*, eram adquiridos em Buenos Aires. Embora a maioria deles viesse diretamente da África, podiam também ser comprados no mercado local ou em outras regiões americanas, ou, então, ser permutados entre as próprias casas ou estabelecimentos jesuíticos, arrendados ou emprestados de outros proprietários. As condições de trabalho dos escravos eram, geralmente, muito ruins, implicando constantes adoecimentos. Como veremos adiante, a despeito da crença de que os escravos pertencentes às ordens religiosas ou a eclesiásticos gozavam de melhores cuidados, se comparados aos escravos de particulares, os escravos dos jesuítas eram afetados por uma série de enfermidades, decorrentes de razões as mais variadas, e que eram tratadas dentro das propriedades da Companhia de Jesus.

Mónica Ghirardi e Liliana Pizzo (2014) estudaram os diários referentes ao atendimento de enfermos prestado por frades betlemitas no hospital San Roque, entre 1762 e 1775, na cidade de Córdoba, tendo constatado que muitos dos pacientes ali atendidos eram escravos ou mestiços. Mesmo que as historiadoras não façam referência aos escravos dos jesuítas, seu estudo é importante para identificar as doenças que acometiam a população da cidade de Córdoba naquele momento, as quais, possivelmente, também atingiram os escravos das propriedades da Companhia de Jesus. Apesar da imprecisão dos diagnósticos, decorrente do conhecimento médico vigente à época, elas identificaram que as enfermidades mais comuns eram aquelas de origem infecciosa (virótica ou bacteriana) e parasitária, muitas delas transmitidas pelo consumo de água ou de alimentos contaminados, por contato com animais hospedeiros ou por contatos físicos entre as pessoas: "Cólera, lepra, febre maligna (malária), tifo, tuberculose, difteria, vermes, sarna, bicheira externa ou interna na cabeça ou nas extremidades; diferentes tipos de feridas em várias partes do corpo, com sintomas de febre e especificamente a chamada de "febre gálica" (Ghirardi, Pizzo, 2014, p.145).[1]

Já para Dora Celton et al. (2020, p.121), na Córdoba do século XVIII, as enfermidades mais comuns eram

> a varíola, disenteria, tuberculose, gastroenterite e pneumonia. Em Córdoba, as mulheres ... sofriam de doenças venéreas, vermes, pleurisia, gangrena, hérnias, fraturas ósseas e hidropisia, entre outras. ... morriam de tuberculose, febre puerperal e venérea e os homens estiveram mais expostos à sífilis e tuberculose.

Para Jorge Troise Melean (2012, p.59), as doenças mais comuns que acometiam os escravos dos jesuítas no período imediatamente posterior a 1767 eram o bócio, a ciática, a demência, a epilepsia, a gastroenterite, a paralisia, a sífilis e a varíola. A grande incidência de doenças em Córdoba, entre os séculos XVI e XVIII, segundo Dora Celton, decorria, principalmente, das vulnerabilidades sanitárias e da escassez de alimentos. A febre tifoide, por exemplo, que era causada pela bactéria *Salmonella typhi*, estava relacionada com o estado alimentar da população e atingia as camadas mais pobres (Celton et al., 2020, p.112).

De fato, entre 1700 e 1749, Córdoba vivenciou 9 anos de secas, 6 anos de tempestades acompanhadas de granizos, 4 anos de pragas nas plantações, 17 anos de encarecimento dos preços das mercadorias e 19 anos de epidemias. Em apenas 16 anos não houve a incidência de pelo menos um dos fenômenos. Entre 1707 e 1720, em apenas três anos não foram registradas epidemias, sendo que o período de maior descenso populacional foi o de 1717 a 1720 e, em especial, 1718, tido como "o ano da peste". A epidemia de varíola que grassava na região teria se originado no desembarque de escravos enfermos no porto de Buenos Aires e se disseminado por Assunção, Vila Rica e Tucumán, chegando até o Alto Peru (Rojas, 2017, p.15). A cidade foi duramente atingida, sendo que, segundo Arcondo (1993, p.68-73), há registros da incidência de peste (bubônica), gota, catarro e sarampo na cidade. Dois outros momentos são também identificados pelo autor como de grave crise sanitária em Córdoba, embora ele reconheça que tenha havido outros episódios menores. O primeiro em torno de 1729, e, o segundo, entre 1741 e 1744. A crise sanitária de 1729-1731 afetou, principalmente, os párvulos, crianças com até 12 anos (Arcondo, 1993, p.75).

Neste texto, apresentamos e discutimos os gastos tanto com o restabelecimento da saúde quanto com o enterramento dos escravos falecidos em decorrência de enfermidades[2] a partir dos registros feitos no *Libro de cuentas*,[3] mais especificamente no *Libro del Oficio del P. Procurador de Provincia donde se ponen las entradas, y gastos de Almacén segun el orden del P. Visitador, y Vice Prov. Antonio Garrida por março del año de 1711* (adiante LCO), referentes ao período de 1711 a 1745. Os dados obtidos a partir da análise deste relatório contábil[4] são cruzados com as informações que constam nos *Memoriais dos provinciais*[5] e nas *Cartas ânuas*.[6] Vale lembrar que a Procuradoria da Província Jesuítica do Paraguay, também chamada de Ofício, era a instância responsável pela administração temporal da estrutura administrativa da província, do Noviciado de Córdoba e da Estância de Santa Catalina, propriedade rural responsável por seu abastecimento.

## Gastos dos jesuítas com doenças e mortes de seus escravos

Na Córdoba de meados do século XVIII, um escravo sadio e em plenas condições laborais custava em torno de 300 pesos. Esse valor equivalia a 150 vacas ou a 45 mulas mansas, a 30 jarros de vinho ou a 300 varas de tecido, ou, ainda, ao pagamento de salário de 6 anos de prestação de serviço de um experiente trabalhador contratado por um salário prefixado, que, na documentação, aparece sob a denominação de conchavado. O preço pago por um escravo era bastante alto, e perdê-lo significava um prejuízo econômico considerável, razão pela qual manter os cativos saudáveis e produtivos era uma preocupação constante de quem tinha, por função, deles cuidar.

Um caso que demonstra que havia um investimento considerável de recursos na compra e tratamento de saúde dos trabalhadores escravos, resultando, ao final, em prejuízo econômico, aconteceu em Córdoba, em 1719 e 1720, e envolveu Juancho Foranzos. Datado de julho de 1719, o registro da compra de Juancho pelo procurador da província,[7] possivelmente na própria cidade de Córdoba, informa: "Por um negro como de 20 anos chamado Juancho que comprei de Dona Catalina Foranzos" (LCO, 1711-1745, folha 181v).

Em abril de 1720, assim como os demais “escravos da Província”, denominação dada aos escravos ligados ao Ofício e que respondiam diretamente ao procurador da província, Juancho recebeu roupas no valor de 1 peso: “A Juancho Foranzos escravo 1 vara de baeta” (LCO, 1711-1745, folha 191). Um mês depois, Juancho aparece recebendo a costumeira dieta dos enfermos, composta de carneiro, aves e pão, no valor de 1 peso e 4 reales:[8] “A 6 de maio, carneiro, aves e pão a Juancho Foranzos, escravo da Província enfermo” (folha 192v). Em julho, a dieta se repete: “Para Juancho Foranzos escravo enfermo, carneiro, pão e aves 8 pesos e um real” (folha 195v). E, em agosto de 1720, morre Juancho, 13 meses depois de ser comprado: “Por 5 varas de tecido para mortalha de Juancho Foranzos, escravo – 5 pesos” (folha 196).

As fontes pesquisadas não trazem mais detalhes sobre a enfermidade de Juancho, não sendo possível saber se já se encontrava doente quando de sua compra ou se adoecera depois de se encontrar trabalhando em uma propriedade dos jesuítas. Tampouco nos informa se houve algum processo judicial movido contra sua antiga dona ou não. O que se sabe é que, em 1720, Córdoba experimentava os efeitos de uma epidemia, e, do ponto de vista econômico, a compra do referido escravo pelo Ofício da Província acabou por causar grande prejuízo.

Para nos aproximar da dieta alimentar do escravizado Juancho é preciso acessar os conhecimentos médicos então vigentes. Tomando por base o *Libro de cirugía*, de 1725 (Fleck, 2022), que sistematiza procedimentos curativos adotados pelos missionários no tratamento dos indígenas enfermos das doutrinas atendidas pelos jesuítas, é possível reconstituir a cultura médica da época, bem como a apropriação de saberes nativos pelos missionários. Mesmo que o manual não se refira especificamente ao tratamento de escravos, é possível que as informações nele contidas tenham sido utilizadas indistintamente no tratamento de doentes que viviam nas propriedades da companhia e fora delas. Também a *Materia médica misionera*, de 1710, escrita pelo irmão jesuíta Pedro Montenegro, pode ter tido suas receitas e recomendações aplicadas pelos encarregados do tratamento dos escravizados enfermos que trabalhavam nas propriedades da Companhia de Jesus.[9]

Assim como a carne de aves, também a carne de carneiro, tida como de fácil digestão, apresentava propriedades terapêuticas. No *Libro de cirugía*, encontramos descritos o uso da carne de carneiro na composição de cataplasmas a fim de tratar dores, e “sossegar os espíritos”, usando-se o pulmão do animal ainda quente sobre a cabeça do doente. Também se indicava o uso do fígado de carneiro no tratamento de olhos vermelhos, de sua vesícula em enfermidades dos ouvidos, de sua carne em fluxos de sangue pelo nariz e pela boca, das tripas e da gordura para desobstrução do ventre. Com a carne da cabeça do animal podia ser preparado remédio para curar diarreia hemorrágica, e com os miúdos bem cozidos podiam ser realizadas purgas. A carne era ainda indicada no tratamento de febres coléricas e sanguíneas e na febre terçã (Fleck, 2022, p.450).

Em relação à utilização de carne de aves na dieta de enfermos, o *Libro de cirugía* aponta para o uso diferenciado da carne de frango, de galinha e de galo, dependendo da doença e das condições físicas do doente. A carne de frango era utilizada no preparo de caldos oferecidos a enfermos bastante debilitados, enquanto o caldo de frango era usado para corrigir a “compleição depravada”. Se os cérebros das galinhas, consumidos com vinho, eram tidos como remédio contra as picadas de víboras, os cérebros dos galos eram indicados para a “fraqueza de estômago”. Já o caldo de um galo velho podia, entre as várias aplicações sugeridas, ser usado para purgar o ventre, dores nas articulações, contra febres e flatulência (Fleck, 2022, p.444-576).

Pelos registros contábeis, constata-se que o Ofício forneceu alimentos e roupas ao escravo Juancho durante sua enfermidade. A necessidade de cuidar dos enfermos foi relatada por Thomas Falkner (1835, p.36), médico cirurgião inglês que ingressou, posteriormente, na companhia e realizou sua formação em Córdoba no final da década de 1730:

> A varíola introduzida neste país pelos europeus causa maiores estragos entre eles [peguenches e picunhes] do que a peste, devastando povoados inteiros com seus efeitos maléficos. 'Essa doença é muito mais fatal para essas pessoas do que para espanhóis ou negros, devido às roupas grosseiras, má alimentação, falta de remédios e cuidados necessários' (destaques nossos).

Na documentação relativa à gestão da província, localizamos, no entanto, várias ordens emitidas pelos superiores, nas quais a situação em que viviam os escravos é descrita, contradizendo as palavras de Falkner, como se pode observar na passagem sobre a Estância de Santa Catalina, estabelecimento rural ligado à Procuradoria da Província:

> 'Os ranchos onde moram os escravos estão muito maltratados e com pouco ou nenhum abrigo; alguns deles expostos a intempéries e tempestades e ao adoecimento dos escravos;' Ordeno que se arrumem o melhor possível e que se construam outros novos ranchos (Memorial del P. Provincial Blas de Silva en la visita de 7 de mayo para la Estancia de Santa Catalina citado em Piana, Cansanello, 2015, p.43; destaques nossos).

A falta de uma estrutura adequada para o alojamento dos escravos continuou sendo um problema na mesma estância por um longo tempo, agravado pelo aumento do número de trabalhadores cativos, como indica a ordem de 1741: "Suspenda-se a obra da sacristia enquanto estiverem sendo construídos os ranchos que a gente habita" (Memorial del P. Provincial Antonio Machoni para la Estancia de Santa Catalina en la visita de 17 de marzo de 1741 citado em Piana, Cansanello, 2015, p.336). O problema parece ter persistido, fazendo com que o próximo padre provincial tenha ordenado em 1746: "Prossiga-se a obra da rancharia até concluí-la e se edificará e cubrirá com telha o que se queimou próximo da padaria" (Memorial del P. Provincial Bernardo Nusdorffer para la Estancia de Santa Catalina en la visita de 6 de febrero de 1746 citado em Piana, Cansanello, 2015, p.369).

A fim de melhor avaliar o impacto das doenças sobre os escravizados que trabalhavam junto ao Ofício da Província Jesuítica do Paraguay e à Estância de Santa Catalina, apresentamos no Quadro 1 seu número total entre 1718 e 1745:

**Quadro 1: Número de escravos da Província Jesuítica do Paraguay e da Estância de Santa Catalina, 1718-1745**

| Ano | N. de escravos | Ano | N. de escravos |
|---|---|---|---|
| 1718 | 366 (Santa Catalina) | 1724 | 297 (Estância e cidade) |
| 1720 | 18 (Ofício)<br>234 (Santa Catalina) | 1728 | 320 (Estância) |
| 1721 | 20 (Ofício)<br>244 (Santa Catalina) | 1734 | 355 (total do Ofício – cidade e estância) |
| 1723 | 20 (Ofício) | 1745 | 317 (estância) |

Fonte: LCO (1711-1745).

Mesmo que os dados populacionais que constam no *Livro de cuentas del Oficio* sejam escassos e, por isso, não permitam avaliar a variação numérica da população de forma mais detalhada, chama a atenção a diminuição do número de escravos na Estância de Santa Catalina no período de 1718 a 1720 e de 1728 a 1745, acompanhada do aumento dos gastos com enfermos ou mortalhas.

Os enfermos que estavam sob os cuidados do procurador da província recebiam porções de carne de carneiro na sua dieta alimentar, cumprindo preceito médico. Em junho de 1717, o Ofício registrava que, devido à ocorrência de enfermidades, recebeu carneiros da Estância de Santa Catalina: "Vieram 20 carneiros para os enfermos, sendo que lhes era dado todos os dias, por ordem do médico, um pedaço de carneiro – 20 pesos" (LCO, 1711-1745, folha 24v). Com relação aos pagamentos de medicamentos pelo procurador da província, várias foram as ocasiões em que foram destinados aos chamados escravos da província, como em agosto de 1712: "No dito dia 100 pesos a Juan Pasqual pela assistência de um ano aos enfermos da rancharia" (folha 146v).

Considerando que um trabalhador contratado do Ofício ganhava entre quatro e seis pesos por mês, como indicado no *Libro de cuentas*, os jesuítas, como se pode constatar, valorizavam bastante o trabalho realizado por esse profissional médico. Chama-nos a atenção, entretanto, o fato de a Procuradoria da Província ter efetuado pagamentos a pessoas de fora da Companhia de Jesus que prestaram atendimento aos enfermos. Uma explicação para essa prática pode ter relação com a falta de religiosos qualificados ou com o grande número de doentes que demandavam a contratação de pessoas habilitadas nas artes de curar. As fontes não nos informam se a rancharia tinha ou não algum religioso encarregado do cuidado dos escravos, tampouco se foi cogitado o deslocamento de um irmão ou padre capacitado de outra propriedade jesuítica para atendimento dos enfermos.

Na relação de gastos feitos em julho de 1717, encontramos a informação de que alguns doentes eram levados à Córdoba para receber atendimento médico: "Dei a um negro enfermo que veio da estância para curar-se e veio sem cobertor ou coisa com que cobrir-se. Dei a ele 4 varas de lã grossa para seu cobertor" (LCO, 1711-1745, folha 170v). Dessa anotação, deduz-se que, quando enviados para tratamento na cidade, os escravos deveriam levar consigo seu cobertor, e, por isso não ter ocorrido, foi alvo de registro.

As despesas com a botica do Colégio Máximo de Córdoba[10] foram constantes no decorrer dos anos, como a que consta sob a rubrica de Gasto Extraordinário em dezembro de 1714: "Por 75 pesos que se deviam à Botica" (LCO, 1711-1745, folha 156v). Na relação de gastos de agosto de 1717, encontra-se o registro do pagamento de 44 pesos à botica por medicamentos dados aos escravos da estância que haviam se dirigido à cidade para receber tratamento: "44 pesos que se pagaram ao Ir. Boticário por vários remédios e medicinas que havia dado a vários negros enfermos que haviam vindo para curar-se da estância de Santa Catalina" (folha 171).

Na relação de gastos de novembro de 1719, encontramos o registro do pagamento de uma série de medicamentos e, também, de alimentos que compunham a dieta de Pacheco e de sua mulher: "60 pesos em 'fórmula magistral de espinheiro' e outros medicamentos, pão, carneiro etc." (LCO, 1711-1745, folha 189). Mesmo que não se tenha a informação de que os enfermos mencionados pelo nome eram escravos, o registro nos dá uma ideia do tratamento a que os doentes eram submetidos.

Apesar de irmãos e padres jesuítas terem se dedicado ao melhor conhecimento das virtudes medicinais de plantas nativas da Província do Paraguay e registrado sua aplicação em manuscritos que circularam entre colégios, residências, estâncias e reduções, "caixas com remédios" aparecem com frequência nas listas de embarque de navios que vinham da Europa para a América. Instrumentos médicos, bisturis e lancetas também podem ser encontrados nas listas, conforme estudo realizado por Corinna Gramatke (2019, p.228).

Em 1720, encontramos o registro de pagamento feito a um boticário não jesuíta pelo tratamento dado a Pacheco e a sua mulher e, na sequência, encontramos os gastos com enfermos do noviciado acometidos de varíola:

> A D. Pedro Gonzalez, Boticário e médico, que empenhou-se para a cura de Pacheco e de sua mulher se lhe deu para sua viagem 12 pesos em moeda, duas camisas de linho de Ruão com arandelas de cambraia, duas calças, uma libra de polvilho e 3 libras que se lhe haviam dado antes – 35 pesos; por 12 reales para pão aos enfermos de varíola aos enfermos do noviciado de baixo – 1 peso e 2 reales (LCO, 1711-1745, folha 179-179v).

O Ofício da Província continuou tendo gastos com a dieta de enfermos em 1720, como se pode constatar nos registros referentes a setembro: "A 23, a Hilário e sua mulher, escravos da Província, enfermos, pão, carneiro e aves 4 pesos e 3 reales" (LCO, 1711-1745, folha 197v); e a novembro: "Para a mulher de Romano, enferma, aves, carneiro e pão 4 pesos e 2 reales" (folha 199v). Os gastos com dieta especial se mantiveram em 1721, sendo que, no mês de janeiro, há menção ao pagamento feito a uma mulher, Josepha Olmos, pelo atendimento de enfermos:

> Para Thomasito Alvarez, escravo da Província, enfermo, em aves, carneiro, pão etc. – 5 pesos e 3 reales ..., a Phelipa mulher de Romano, enferma, em aves, carneiro, pão etc. – 1 peso ..., a Dona Josepha Olmos, por sua assistência aos enfermos de nossa Rancharia – 12 pesos. ... para Thomasito, enfermo, 3 pesos (LCO, 1711-1745, folha 201, 201v).

O pagamento a Josepha Olmos chama a atenção por vários motivos. O primeiro deles diz respeito ao fato de que, mais uma vez, foi feito o pagamento a alguém de fora da Companhia para atendimento dos enfermos da rancharia; e o segundo, por Josepha Olmos ser mulher. Sabe-se que os jesuítas mantinham cuidados especiais para distanciá-las dos homens, independentemente de sua condição social, o que nos leva a perguntar: qual a formação de Josepha Olmos e qual sua relação com a companhia, considerando que lhe foi confiado o tratamento dos escravos enfermos?

Em fevereiro de 1721, seriam mencionados os seguintes gastos: "14 para os três enfermos de nossa rancharia com carneiro, aves etc. 5 pesos moeda – 5 pesos ... para Romano e a mulher de Pedro Estanciero enfermos 12 reales moeda – 1 peso e 4 reales" (LCO, 1711-1745, folha 202v). No mês de abril de 1721, encontramos os registros referentes ao pagamento feito à parteira que atendeu a mulher de Romano e à compra de roupas para outra parturiente e de outros gastos com saúde. Pelos registros contábeis, a mulher de Romano recebeu cuidados durante três meses, muito possivelmente, devido a algum problema de saúde relacionado com a gravidez:

> À parteira da mulher de Romano 7 pesos e 2 reales; e pão, carneiro e aves à parturiente 2 pesos; e 3 varas de baeta para mantilhas – 12 pesos ..., a Catalina escrava de Thomasito Alvarez em seu parto 5 varas de lã grossa para cobertores, 3 varas de baeta para mantilhas e 3 pesos em moedas para pão, aves e carneiro – 10 pesos ..., ao ceguinho Francisco escravo da Província e a Isabel escrava enferma 10 pesos em roupa – 10 pesos (LCO, 1711-1745, folha 204).

A análise que realizamos dos livros contábeis dos jesuítas indica que os gastos com parteiras para atendimento de mulheres escravas eram bastante incomuns. Isso nos leva a supor que eles recorriam a atendimentos especializados somente quando surgiam problemas antes ou durante o parto. Nos outros momentos, muito provavelmente, as parturientes eram atendidas por outras mulheres de sua condição, experientes na arte de partejar. Há que referir que o já mencionado *Libro de cirugía* trata, em um de seus capítulos, dos partos, bem como dos cuidados que deveriam ser observados no período que se seguia a eles. Nesse capítulo, são mencionados os remédios que deveriam ser utilizados para facilitar os partos, aqueles que provocavam a menstruação, os que davam movimento ao feto, como os medicamentos esternutatórios, os vomitórios, que produziam movimentos internos, além de diversos chás e alguns medicamentos incomuns, como beber um copo de urina do marido ou usar excrementos de animais para parar o fluxo sanguíneo depois do parto (Fleck, 2022, p.429-435).

Com relação aos gastos com roupas dos escravizados dessas propriedades jesuíticas, os tecidos eram entregues na entrada do inverno, visando à proteção dos corpos dos trabalhadores submetidos aos rigores climáticos, mas isso podia acontecer em outros momentos, quando as roupas se encontravam gastas, justas ou curtas demais ou quando havia algum evento especial, como casamentos e nascimentos.

Considerando que, em 1721, os "escravos da Província" eram em número de vinte, constata-se que o percentual de doentes naquele momento era bastante alto, como demonstram os diversos gastos tidos com eles durante o ano. Em relação ao mês de maio, tem-se a informação de que "deu o Padre Simon Baylina para Romano enfermo 10 reales para pão, carneiro etc. 1 peso e 2 reales" (LCO, 1711-1745, folha 205v). No mês seguinte, o enfermo recebeu um par de sapatos, algo que raramente acontecia, pois não era um item entregue aos escravos pelos jesuítas: "A 23, a Romano enfermo um par de sapatos, e ao mesmo, a Pedro Estanciero, Pedrito Albornoz e Antonio 11 varas de roupa – 12 pesos" (folha 206v). Enquanto em julho: "A 3, para Pedro Estanciero enfermo 14 reales para pão, carneiro, aves etc. – 1 peso ..., para Pedro Estanciero enfermo 5 reales ..., à enferma Andrea para pão, carneiro etc. 3 pesos moeda" (folha 207, 208).

Quanto à entrega de sapatos aos escravos, o padre provincial Agustín de Aragón deixou registrado, em seu memorial de 1669-1672, que essa prática incomum estava liberada, desde que aqueles que os recebessem fossem principais, isto é, possuíssem liderança sobre os demais, constituindo-se em um pequeno privilégio que os diferenciava socialmente dos outros escravos. Os nomes relacionados no registro aparecem constantemente no *Libro de cuentas* do Ofício, levando e trazendo bens à Estância de Santa Catalina, sinal de que gozavam de prestígio junto ao procurador:

> Para as pequenas coisas ordinárias que geralmente se pedem para um enfermo, como um copo de vinho, um pouco de azeite, pomada, um pouco de açúcar ou outro doce, também a um negro ou dois dos principais poderá dar sapatos, calções, camisa ou coisas equivalentes. Aos enfermos do nosso serviço, o que precisarem[11] (Memorial 1669-1672. Del P. Provincial Agustín de Aragón citado em Piana, Cansanello, 2015, p.517).

Em outubro de 1721, o Ofício teve dois gastos consideráveis com a botica, um deles relativo aos cuidados com um escravo cego e outro com a compra de medicamentos:

> A 12, a Bartholito, o Boticário, por sua assistência ao negrinho cego enfermo e a outros enfermos da Província um monte de peles de lebre e forro de juta – 7 pesos …, a 16 de outubro paguei à Botica 35 pesos e 5 reales que foi o total dos medicamentos que até este dia havia dado à estância e a este Ofício – 35 pesos (LCO, 1711-1745, folha 211).

Em novembro de 1721, encontramos a menção à dieta indicada para Pedro Estanciero, "escravo nosso enfermo", que deveria receber pão, aves e carneiro (LCO, 1711-1745, folha 213). Cabe ressaltar que, nos registros da Procuradoria da Província, temos não apenas acesso ao nome dos escravos enfermos, mas também à duração de seu tratamento, sendo que alguns receberam cuidados por vários meses, o que possibilita o acompanhamento da evolução da condição da saúde de cada trabalhador.

Neste mesmo ano, o Ofício comprou grande quantidade de enxofre, não indicando o uso que seria feito dele. Sabe-se que o produto podia ser utilizado na vitivinicultura, na fabricação de pólvora e, também, na composição de receitas para o tratamento de certas doenças.[12] Os livros de contas não indicam a sua utilização como medicamento, mas deduz-se que possa ter sido usado com esse fim, uma vez que a botica dos jesuítas de Córdoba já o havia adquirido em outro momento, como se pode constatar na compra feita pela Procuradoria da Província: "Não avisa tampouco o preço do enxofre, e calculado pelo que se enviou à Botica se lhe abonou a 3 pesos e 4 reales a arroba" (LCO, 1711-1745, folha 37).

Em 1722, são mantidos os gastos com enfermos, e, mais uma vez, encontramos registro de pagamento feito à já citada Josepha Olmos. Em fevereiro, encontramos registrada a seguinte despesa: "Gastei com os enfermos de nossa rancharia 7 pesos" (LCO, 1711-1745, folha 215v). E, em abril, novamente a menção ao atendimento dos enfermos:

> De nossa Rancharia este mês 13 pesos em moeda ...; por 7 frangos, 15 galinhas e 7 quartos de carneiros para os enfermos de nossa Rancharia por 6 pesos e 2 reales ..., a Dona Josepha de Olmos por assistir aos ditos enfermos 5 varas de sarja e uma vara de [ilegível] para saia de sua filha – 16 pesos e 4 reales ..., a 27, para nossos enfermos 28 pesos moeda (LCO, 1711-1745, folhas 217, 217v).

Nos registros relativos ao mês de maio de 1721, lê-se: "Aos enfermos, pão, aves e carneiro – 2 pesos" (LCO, 1711-1745, folha 218). E, nos de outubro, há a seguinte menção: "Com os enfermos da Rancharia para aves etc. 5 pesos, e um peso para a médica Dona Josepha – 6 pesos ..., a 12, à botica 2 arrobas de erva 4 pesos ..., a 23, à Botica uma arroba de erva" (folha 222v). Como se pode constatar nesta última passagem, Josepha Olmos é apresentada como médica, e sua remuneração se deve ao atendimento prestado a escravos enfermos da rancharia. Ela seguramente não era médica; no entanto, seus conhecimentos práticos de medicina deviam ser valorizados pelos jesuítas de Córdoba, que a contratavam em determinadas situações.

Os gastos com saúde em fevereiro de 1723 foram altos, destacando-se o pagamento feito a um médico português: "A D. Joseph o médico Português, pela cura da ferida do Padre Mestre de Noviços e pela cura da negrinha Isabel de Santa Catalina dei-lhe 30 pesos em moeda" (LCO, 1711-1745, folha 225v). No mês seguinte, aparecem discriminados os gastos com a botica: "A 17 à Botica 1 libra e 1/2 de polvilho a 4 pesos e 4 reales" (folha 226), e, em setembro, encontramos nova menção a gastos com a saúde de uma escrava: "À Rosa mulher de Pancho Fernandez escrava da Província para seu vestuário, e, em sua última enfermidade, 21 pesos e 5 reales" (folha 232). Em outubro, encontramos a informação: "À botica, 12 varas de baeta ...; 12 varas de lenços; 12 varas de barbante este a 10. 33 varas e 1/2 de fita a 6 reales cada e 14 varas da dita fita a 4 reales e uma libra de anil a 4 pesos ... são – 62 pesos e 6 reales" (folha 233). E, em novembro, se repete a menção a despesas com dieta para enfermos: "Aves e carneiro para os enfermos da rancharia do Noviciado e pão etc. 7 pesos moeda" (folha 233v). Em 1724, os gastos da procuradoria foram feitos com dietas de enfermos (aves, pães e carneiros), os quais somaram 5 pesos, e com a botica, no valor de 80 pesos, 1,5 real (folhas 237-242). Em abril de 1724, houve o pagamento da "Parteira que assistiu ao parto de uma morena da Província 4 varas de baeta – 4 pesos" (folha 238).

Em 1728, houve elevadas despesas com enfermos, sendo que, em junho daquele ano, aparecem relacionados gastos "por 4 pesos para umas Morenas enfermas do Noviciado ..., Item por 8 varas de roupa a uma escrava da estância que veio enferma ..., 1 peso moeda para uma escrava enferma" (LCO, 1711-1745, folha 261)*;* em dezembro: "2 pesos e 4 reales a um Escravo Enfermo ... 4 reales aos enfermos" (folha 265).

No ano seguinte há somente um registro no valor de 99 pesos, gastos pelo Ofício com a botica (LCO, 1711-1745, folha 266v), o que não se observa em 1730, ano em que houve várias despesas com escravos enfermos. Entre elas, está a contratação, em julho, de uma indígena para cuidar da filha de uma escrava: "Por 25 pesos que dei a uma Índia contratada por um ano para que crie a filhinha de Juancho Pablo, por estar sua mãe muito enferma" (folha 273v). Cabe notar que o valor pago à indígena está muito abaixo da remuneração dos demais trabalhadores livres contratados, que recebiam entre 40 e 55 pesos por ano, conforme registros de gastos no *Libro de cuentas del Oficio* que analisamos.

Já em agosto de 1730, foram gastos 10 pesos, 2 reales "para a cura de Romano ... e juntamente para a da mulher de Juan Pablo" (LCO, 1711-1745, folha 274). A documentação revela, ainda, que em setembro daquele ano foram contratados pelo Ofício alguns trabalhadores para realizar a função desempenhada por Romano durante sua "convalescença": "A uns moços que me ajudaram enquanto Romano estava enfermo – 6 pesos" (folha 274v). Nos registros relativos ao mês de outubro de 1730, encontramos o pagamento de uma purga e de um sangrador: "Na enfermidade de Romana 3 pesos por uma purga, 12 reales em pão, carneiro, um peso ao Sangrador" (folha 275v).

O tratamento médico nos séculos XVII e XVIII embasava-se, principalmente, nos pressupostos da teoria humoralista, tributária das ideias gregas antigas que viam a doença como consequência de desequilíbrios nos quatro humores básicos (sangue, fleuma, cólera e melancolia). A utilização da sangria visava, assim, reduzir a febre por meio da retirada do sangue quente. Este procedimento consistia em "incisão ou corte artificialmente feito na veia para evacuar todos os humores" (Fleck, 2022, p.340), sendo que o *Libro de cirugía* (Fleck, 2022, p.346-652) apresenta uma longa lista de doenças para as quais era indicada, dentre as quais destacamos erisipela, dores nas costas, convulsões, doenças nos olhos e ouvidos, dores de dente, disenteria, hemorragias depois do parto, úlceras e bubões. Lamentavelmente, no *Libro de cuentas del Oficio* não aparece mencionada a doença que se pretendia curar com a aplicação de uma sangria em Romana.

Mas havia, também, os que consideravam a teoria miasmática, que sustentava que a doença resultava de nuvens de gases tóxicos que emanavam de corpos em decomposição, da vegetação ou de outro material orgânico. Para limitar a propagação dos miasmas, costumava-se enterrar profundamente os corpos no solo para evitar as emanações tóxicas (Jackson, 2014, p.95). Para os defensores dessa teoria, os "ares pútridos" causavam o adoecimento das pessoas, concepção que pode ser observada na *Ânua* de 1730-1735, na referência feita ao Noviciado de Córdoba em 1731:

> Decaiu muito o estado temporal desta casa devido às más colheitas dos anos anteriores e à epidemia do ano de 1731, que consumiu 28 dos nossos escravos negros e deixou tão debilitados os demais, que apenas houve peões para cultivar a terra. Procurou-se remediar tantos males através de uma novena oferecida ao Apóstolo das Índias e, ao final dela, caiu do céu uma chuva copiosa, a qual reduziu em grande medida a atmosfera ardente e insalubre de modo que a epidemia terminou em pouco tempo (Cartas anuas de la Provincia del Paraguay de la Compañía de Jesús desde el año de 1730 al año de 1735 citadas em Salinas, Folkenand, 2017, p.222).

Em 1731, a Procuradoria da Província registrou um aumento considerável nos gastos com a saúde de seus escravos, aos quais se somaram os valores investidos na reposição da força de trabalho que havia sido vitimada pelas epidemias. Isso pode ser observado no registro de março de 1731, relativo à Estância de Santa Catalina: "2432 pesos e 4 reales que custaram 12 negros para a Estância, valor que levou o Padre Provincial a Buenos Aires" (LCO, 1711-1745, folha 277v). O preço médio de cada escravo girou em torno de 200 pesos, um valor bastante abaixo daqueles praticados em Córdoba em outros períodos. Mesmo que não esteja registrado no livro de contas, esse número relativo à compra de escravos foi, de acordo com a carta ânua de 1720-1730, muito maior:

> A estância teve seus prejuízos; no entanto, adquiriu 25 escravos para substituir outros tantos que sucumbiram à varíola. Todos foram atingidos e um quarto foi transformado em hospital, sendo que serviram de enfermeiros os próprios padres, com muita humildade e caridade (Cartas ânuas de la Provincia del Paraguay de la Compañía de Jesús desde el año 1720 hasta el mes de octubre del año 1730 Enviadas al muy reverendo padre vicario general de la misma Compañía, P. Francisco Retz citadas em Salinas, Folkenand, 2017, p.117).

No mês de agosto de 1731, há o registro da despesa no valor de 16 pesos e 4 reales com cuidados prestados ao escravo Romano "em sua grave e longa enfermidade, em reses e remédios, 16 pesos e 4 reales" (LCO, 1711-1745, folha 279v), valor que aumentou em setembro, quando foram gastos "em remédios para a estância em várias vezes 22 pesos" (folha 279v), enquanto, em outubro, houve a necessidade de atendimento especializado:

"A Juan Pasqual, médico, por haver assistido no Noviciado e a vários negros enfermos" (folha 280). Em 1732 e 1733, a epidemia parece ter sido controlada, uma vez que o único registro de gastos com saúde de escravos se refere ao pagamento de um cobertor destinado a um trabalhador enfermo no mês de junho de 1733: "Ao negro enfermo, um cobertor, 8 pesos" (folha 291v).

Nos registros de gastos do mês de junho de 1733, há a informação de que um escravo tivera os dedos cortados; contudo, nem as circunstâncias nem seu nome são mencionados no documento, mas é plausível supor que tenha sido uma punição por algum crime cometido, possivelmente, roubo: "Ao negro que se lhe cortaram os dedos 9 varas de roupa para vesti-lo ... e confecção 2 pesos" (LCO, 1711-1745, folha 292). Em setembro de 1733, os registros no livro de contas da Procuradoria da Província indicam gastos com outro escravo: "7 pesos que se gastaram na enfermidade de Juan Pablo" (folha 293v).

Não se sabe se, por esquecimento ou se pela inexistência de gastos, mas, no período de 1734 a 1739, o Ofício da Província não registrou quaisquer despesas com atendimento de enfermos para além daqueles ordinários. Quase ao final de 1739, em novembro, encontramos uma menção à aquisição de remédios: "10 pesos em remédios que levou o Padre Florentin para a Estância" (LCO, 1711-1745, folha 324v), e, posteriormente, em dezembro, quando foi registrado que Juan Pablo recebia mais uma vez a dieta costumeira dos enfermos: "Por 1 peso para galinhas a Juan Pablo enfermo" (folha 324v).

O próximo registro data de junho de 1740 e se refere a gastos da Procuradoria com as provisões da botica*:* "2 varas de lenços à botica; 1/2 alqueire de farinha para os pães que se dão no Colégio aos nossos enfermos e Negrinhos" (LCO, 1711-1745, folha 327v). Já em setembro daquele ano, houve o pagamento à botica de "12 pesos e 4 reales, que valia uma jarra de vinho, que o Ir. Boticário comprou" (folha 329).

O vinho, vale lembrar, possuía inúmeras aplicações terapêuticas e, como indicado no *Libro de cirugía*, era utilizado no preparo de medicamentos, em sangrias e purgas, em cataplasmas, na dissolução de medicamentos, na composição de bálsamos e em tantas outras prescrições medicamentosas. Também o irmão jesuíta Montenegro (2009, p.193), em sua *Materia médica misionera*, reconhece a importância do vinho em diversos preparados, tais como o que deveria ser empregado contra o veneno de animais peçonhentos: "O ciclamen ... bebido seu pó com vinho, é o único remédio contra venenos mortais".

Dado o cenário sanitário de Córdoba durante 1742 e 1743, a Procuradoria teve gastos consideráveis com saúde nesse período, como o pagamento de alguns itens e dos honorários de alguns encarregados das artes de curar. Em julho de 1742, por exemplo, gastou-se, na estância da procuradoria, com "várias coisas para assistência e com presentes para os enfermos" (LCO, 1711-1745, folha 338), e, em outubro, encontramos o seguinte registro: "Este mês de outubro gastei na Estância 4 pesos em biscoitos para os enfermos ..., uma jarra de aguardente de 18 pesos" (folha 339v-340v). Já em novembro de 1742, houve a seguinte menção: "Dei este mês à Estância ... várias coisas para os enfermos" (folha 340). Enquanto, em dezembro, foi registrado: "Dei este mês à Estância ..., 1 jarra de aguardente que custou 30 pesos para os enfermos ..., 17 pesos e 3 reales ao que assistia os enfermos; 28 pesos a Moyano, que os curava; 45 pesos à mulher e filhas de D. Luciano Salgado, que os assistiam e curavam" (folha 340v); em outubro de 1743: "100 pesos à botica" (folha 344). Também em dezembro de 1742, há o registro de pagamento de soma considerável por remédios "ao Ir. Enrique Peschke por remédio gasto no Noviciado e em sua Rancharia 93 pesos e 7 reales em moeda" (folha 148).

Nos registros de gastos referidos chama a atenção, mais uma vez, a assistência aos enfermos prestada por leigos encarregados das artes de curar. Considerando que os trabalhos foram pagos, pode-se supor que isso tenha ocorrido devido à falta de jesuítas habilitados para prestar atendimento, e não à caridade de alguns moradores de Córdoba. Deve-se, ainda, conjeturar que a urgência no atendimento e o grande número de enfermos tenham feito com que até os serviços de um chapeleiro e das filhas de um nobre tenham sido requisitados.

Em 1744, há três registros, um deles sobre atendimento de doentes e outros dois sobre o provimento da botica. O primeiro deles dá conta, também, do prejuízo com a morte de um escravo recém-comprado: "E paguei 200 pesos por um Negrinho que morreu em Buenos Aires" (LCO, 1711-1745, folha 345v). Enquanto o segundo, com data de outubro de 1744, menciona o gasto com "1 libra de polvilho ao Ir. Boticário – 3 pesos" (folha 348), e o de dezembro registrou: "Neste mês paguei pelo Noviciado à botica 39 pesos" (folha 348v). Os gastos com medicamentos se mantiveram no ano seguinte, como se pode constatar no registro do pagamento pela província, efetuado em janeiro de 1745, de "40 pesos e 4 reales à botica pela província" (folha 349), e daquele feito em dezembro de 1746: "Dei à Botica 200 pesos pelos gastos em 4 anos ... e paguei à Botica pela Estância 168 pesos, dos quais 100 estão anteriormente registrados" (folha 344v).

Em 1746 e 1747, localizamos cinco pagamentos relativos à aquisição de medicamentos por parte do Ofício, totalizando 117 pesos, 4,5 reales (LCO, 1711-1745, folhas 104v-110). Depois de um longo período sem despesas com a botica, em junho de 1752, e em março e setembro de 1753, respectivamente, encontramos três vultuosos pagamentos: "Pelo que paguei à Botica neste mês – 106 pesos (folha 121); Que em moeda e outros efeitos paguei à Botica – 168 pesos (folha 123v); Paguei à Botica cento e cinquenta e cinco pesos, valor que totalizaram as contas" (folha 125). Já em fevereiro de 1754, há o registro de um valor menor empregado na compra de "vários remédios que se gastaram com os negros do Ofício – 6" (folha 126).

Mesmo que os registros dos gastos que o Ofício da Província Jesuítica do Paraguay realizava com a botica não distinguissem o público atendido, pois podiam se referir tanto aos religiosos quanto "à gente de serviço", é possível perceber que eles consumiam boa parte dos recursos econômicos disponíveis para a manutenção da saúde dos trabalhadores escravizados. Esses gastos foram proporcionalmente maiores nos momentos de epidemias, mas continuaram sendo realizados também em outros momentos, indicando que o adoecimento dos religiosos e dos trabalhadores era constante e que os cuidados com a saúde, conforme indicado nos memoriais dos superiores, visavam mitigar os efeitos econômicos e seu impacto no desenvolvimento das atividades-fim da Companhia de Jesus. As fontes analisadas demonstram que os escravos da propriedade dos jesuítas na Córdoba do século XVIII foram afetados diretamente pelo contexto sanitário de Córdoba, estando sujeitos à toda sorte de enfermidades e epidemias.

## Considerações finais

A documentação analisada neste artigo permite reconstruir, mesmo que fragmentariamente, as condições de saúde dos trabalhadores escravos ligados ao Oficio da Província Jesuítica do Paraguay e à Estância de Santa Catalina na primeira metade do século XVIII. Uma leitura atenta dos *Memoriais dos provinciais* e das *Cartas ânuas* permite perceber que a jornada extenuante de trabalho dos escravos podia ser, ao lado das doenças comuns do período e da escassez alimentar, uma das razões para seu adoecimento, sendo que os superiores, por diversas vezes, lembraram os administradores quanto aos cuidados que deviam ser a eles dispensados.

Nas estâncias, onde o número de escravos era significativo, fica mais difícil mensurar quais eram suas condições de saúde e das instalações nas quais viviam. Já em relação aos escravizados do Ofício, que eram em número menor, as fontes analisadas revelaram uma alta incidência de enfermidades entre eles, bem como constantes gastos – com vários deles – por longos períodos, durante os quais permaneceram afastados de suas funções laborais ou dedicaram-se a outras por um tempo reduzido. De qualquer forma, o fato de o Ofício ter tido, simultaneamente, três ou quatro escravos doentes em um universo de vinte trabalhadores representava um percentual bastante elevado, se considerado o número total de trabalhadores.

Nas primeiras décadas do século XVIII, a cidade e a região de Córdoba sofreram os efeitos de estiagens, granizos, pragas nas lavouras e epidemias, que se viram acompanhados de um elevado número de mortes. Os trabalhadores das propriedades jesuíticas foram, assim como a população que vivia na cidade, duramente atingidos por essa conjuntura, determinando a destinação de recursos tanto para o atendimento dos doentes ou o seu sepultamento em caso de óbito quanto para a reposição da mão de obra nas estâncias.

A documentação revelou que os doentes eram tratados em enfermarias, e que os acometidos de peste ficavam separados das demais pessoas que recebiam tratamento. Aqueles que necessitavam de algum atendimento especializado eram deslocados até a cidade de Córdoba, o que sugere que as estâncias não contavam com estrutura e pessoal habilitados para o tratamento de enfermos. Ao chegar na cidade, muitos desses doentes eram, em algumas situações, atendidos por leigos ou leigas. Essa informação nos leva a refletir sobre as condições que a Companhia de Jesus tinha, naquele momento, para atender os enfermos de suas propriedades e sobre as razões para não ter assumido seus cuidados, a despeito da bem abastecida botica do Colégio de Córdoba e da experiência que alguns irmãos e padres jesuítas tinham nas artes de curar.

Em relação ao atendimento prestado aos enfermos merece ser, ainda, mencionado que não localizamos registros de escravos cuidadores de outros escravos doentes. A ausência nos registros deve-se, muito provavelmente, ao fato de que as fontes analisadas trazem informações sobre a contabilidade das estâncias, não se detendo, em razão disso, nas formas de atendimento ou naqueles que se dedicavam a esse Ofício. Isso não significa, contudo, que eles não existissem.

Quanto à dieta alimentar dos doentes, o uso de pães, biscoitos, carnes de carneiro e de aves seguia prescrições da medicina vigente e tinha o claro propósito de restabelecer os humores. Entretanto, nem tudo o que era utilizado para recobrar a saúde era registrado nos

*Libros de cuentas* analisados, na medida em que tratados como a *Materia médica misionera* e o *Libro de cirugía* apontam para o uso de saberes locais e de plantas nativas americanas, em substituição aos procedimentos praticados na Europa.

Por fim, a natureza contábil das fontes analisadas limita bastante uma avaliação quanto às formas como os escravos reagiam diante da doença e da morte iminente. Acreditamos, contudo, que, mais do que pacientes passivos diante dos tratamentos a que eram submetidos, eles tenham acionado, sempre que possível, não apenas os conhecimentos ancestrais, mas também a solidariedade, em momentos como os partos, as longas enfermidades e o sepultamento.

## NOTAS

[1] Essa e as demais citações de originais em espanhol têm tradução livre.

[2] As despesas com a compra de tecidos para a confecção de mortalhas, além de apontar para os cuidados em relação ao sepultamento dos escravos falecidos em decorrência das enfermidades, permitem estimar a quantidade de mortos ao longo do período estudado.

[3] Os *Libros de cuentas* são relatórios contábeis em que eram registrados os movimentos de entrada e saída de bens e valores econômicos, pagamentos e recebimentos de serviços prestados. Cada estabelecimento jesuítico realizava os registros contábeis com a finalidade de controle interno. Cabe ressaltar que as análises já feitas dos *Libros de cuentas* não se detiveram nas despesas relativas à manutenção da saúde dos escravizados que os jesuítas mantinham em suas propriedades rurais e nos impactos desses gastos sobre suas finanças, o que confere originalidade à investigação que divulgamos neste artigo.

[4] Entre os autores que têm se debruçado sobre a documentação relativa às propriedades rurais jesuíticas de Córdoba, em especial, sobre os *Memoriais* e os *Libros de cuentas* das estâncias ligadas ao Colégio Máximo, está Carlos Alberto Crouzeilles. Seus trabalhos, apesar de não se deter nos gastos feitos para assegurar a saúde ou o sepultamento adequado dos escravizados, são fundamentais para a reconstituição das atividades produtivas e da vida cotidiana das estâncias mantidas pela Companhia de Jesus nos séculos XVII e XVIII. Sobre esses dois temas, recomendamos ver mais em Crouzeilles (2007, 2013a, 2013b, 2015).

[5] *Memoriales* são os documentos emitidos pelos superiores jesuítas após visita a residências, colégios, estâncias, reduções ou procuradorias contendo instruções acerca da aplicação, na organização cotidiana, das regras e constituições da ordem.

[6] As *Cartas ânuas* eram correspondências anuais enviadas pela província para o generalato em Roma. De gênero edificante, apresentavam os sucessos e as dificuldades da missão desenvolvida nos mais diversos espaços, a aplicação de sacramentos, a morte de missionários. Apesar de apresentar os trabalhos missionários realizados durante o ano, muitas delas reúnem a descrição de atividades de vários anos.

[7] O procurador da Província Jesuítica do Paraguay, além de tratar dos assuntos econômicos e políticos ligados à administração da estrutura provincial, era também o superior dos administradores da Estância de Santa Catalina. Sua função não pode ser confundida com a dos representantes das províncias, eleitos pelas congregações provinciais para participar das congregações gerais ou de procuradores, que também recebiam esse nome. Havia também os procuradores locais, os quais eram responsáveis por casas, colégios, estâncias, missões e províncias. Esse era um cargo de natureza econômico-contábil e era o cargo do procurador da província em questão. A companhia tinha ainda um terceiro tipo de procuradores que trabalhavam por interesse da Companhia de Jesus nas cortes ou em Roma. Por fim, havia ainda procuradores que desempenhavam suas funções nos portos de embarque. Eles eram também procuradores de missões e não podem ser confundidos com os procuradores do segundo tipo (Fechner, 2014, p.32).

[8] Um peso equivalia a 8 reales.

[9] Sobre esses dois tratados setecentistas, recomenda-se ver mais em Fleck (2019, 2022) e Fleck, Biehl (2020).

[10] Sobre a botica do Colégio de Córdoba, veja-se Flachs e Page (2010).

[11] Noutro documento, o padre Agustín não utiliza o termo *principales*, geralmente usado para diferenciar as lideranças indígenas, mas capataz ou benemérito: "A algum Negro Capataz, ou mais benemérito poderá dar-lhe camisa, uns calções de tecido de Quito; uns sapatos" (Preceptos..., 8 sep. 1671).

[12] Sobre o uso do enxofre para o tratamento de dores internas do ventre e do peito, na cura de feridas, da surdez, do "mal de madre", apostemas, sarna e também nos partos difíceis, ver *Libro de cirugía* (Fleck, 2022, p.68).
